# Pancreatic cancer cachexia: a review of mechanisms and therapeutics

**DOI:** 10.3389/fphys.2014.00088

**Published:** 2014-03-03

**Authors:** Carlyn R. Tan, Patrick M. Yaffee, Laith H. Jamil, Simon K. Lo, Nicholas Nissen, Stephen J. Pandol, Richard Tuli, Andrew E. Hendifar

**Affiliations:** ^1^Samuel Oschin Comprehensive Cancer Center, Cedars Sinai Medical CenterLos Angeles, CA, USA; ^2^Department of Medicine, David Geffen School of MedicineLos Angeles, CA, USA; ^3^Department of Medicine, Cedars Sinai Medical CenterLos Angeles, CA, USA

**Keywords:** pancreatic cancer, cachexia, anorexia, catabolism, multimodal therapy

## Abstract

Over the last decade, we have gained new insight into the pathophysiology of cachexia associated with pancreatic cancer. Unfortunately, its treatment is complex and remains a challenge. Pancreatic cancer cachexia is a multifactorial syndrome characterized by uncompensated adipose tissue and skeletal muscle loss in the setting of anorexia that leads to progressive functional impairment. This paper will review the current concepts of pancreatic cancer cachexia, its assessment and pathophysiology as well as current and future treatments. The successful management of pancreatic cancer cachexia will likely require a multimodal approach that includes nutritional support and combination pharmaceutical interventions.

## Introduction

Cachexia is a ubiquitous characteristic of pancreatic cancer and develops in approximately 80% of pancreatic cancer patients during their disease course (Fearon et al., 2006b). Up to one-third die from complications associated with cachexia through immobility, severe respiratory muscle impairment resulting in cardiopulmonary failure, and impaired immunity (Bachmann et al., 2009). Cachexia is a complex metabolic disorder that involves features of anorexia, anemia, and loss of adipose and skeletal muscle mass. In pancreatic cancer patients, it has been associated with reduced physical function, lower response rates to chemotherapy and radiation treatment, and decreased survival (Dewys et al., 1980; Moses et al., 2004; Bachmann et al., 2008, 2009). Pre-operative evidence of cachexia in pancreatic cancer patients has been also associated with worse postoperative outcome after pancreaticoduodenectomy (Pausch et al., 2012).

Although new insights to the pathogenesis of pancreatic cancer cachexia have been gained over the past decade, the underlying mechanisms leading to this syndrome are not fully understood. There continues to be an active search for potential targets and effective treatment. This article reviews the current concepts and management of this clinical dilemma.

## Definition and classification of cancer cachexia

Cachexia has been recognized as a common complication of cancer. In 2011, an international consensus defined cancer cachexia as a multifactorial syndrome characterized by ongoing loss of skeletal muscle mass that cannot be fully reversed by conventional nutritional support and leads to progressive functional impairment (Fearon et al., 2011). Diagnostic criteria include weight loss greater than 5% over the past 6 months, weight loss greater than 2% in individuals with body mass index (BMI) less than 20 kg/m^2^, or evidence of sarcopenia with any degree of weight loss greater than 2% (Table 1). Evidence of sarcopenia is defined as appendicular skeletal muscle index less than 7.26 kg/m^2^ in males and less than 5.45 kg/m^2^ in females determined by dual energy X-ray absorptiometry (DEXA). Based on these criteria, the majority of pancreatic cancer patients have cachexia at the time of diagnosis (Fearon et al., 2006b).

**Table 1:** **Diagnosis of cancer cachexia (Fearon et al., 2011)**.

Weight loss greater than 5% over the past 6 months; or
Weight loss greater than 2% in individuals with BMI less than 20 kg/m^2^; or
Evidence of sarcopenia^*^ with weight loss greater than 2%

* Sarcopenia defined as appendicular skeletal muscle index in males <7.26 kg/m^2^ and in females <5.45 kg/m^2^ determined by DEXA.

Cancer cachexia develops progressively through a spectrum. The international consensus identified three stages of cachexia: precachexia, cachexia, and refractory cachexia (Fearon et al., 2011). Severity is classified based on the degree of depletion of energy stores and body protein mass (using BMI) and the rate of ongoing weight loss. In precachexia, patients demonstrate early clinical and metabolic signs including anorexia and impaired glucose tolerance preceding substantial involuntary weight loss. Patients then develop progressive weight loss and meet the criteria for cachexia as previously defined. Cachexia becomes clinically refractory as a result of progressive cancer unresponsive to therapy. In this stage, there is active catabolism, and patients have worsening physical function with a life expectancy of less than 3 months.

## Assessment of cancer cachexia

Since cachexia is a multifactorial syndrome, its evaluation should involve assessment for various features as summarized in Table 2: anorexia or reduced food intake, catabolic drivers, muscle mass and strength, and functional and psychosocial effects (Fearon et al., 2011). Some of these different characteristics of cancer cachexia have been found to be adverse prognostic indicators. A recent study showed that weight loss (>10% weight loss), reduced food intake (<1500 kcal/day), and evidence of systemic inflammation [C-reactive protein (CRP) > 10 mg/L] identified pancreatic cancer patients with reduced subjective and objective functional ability. Patients with at least two of these components had a significantly worse prognosis (Fearon et al., 2006b).

**Table 2:** **Assessment of cancer cachexia**.

**Areas of assessment**	**Methods**
Reduced food intake/ anorexia	Patients estimate overall food intake
	Third-party assessment of food intake (family member)
	Assess for mechanical factors contributing to reduced intake
Hypercatabolism	Serum CRP levels
	Responsiveness to treatment and rate of disease progression
Muscle mass and strength	Cross-sectional imaging with CT or MRI
	DEXA: appendicular skeletal muscle index
	Anthropometry: mid-upper-arm muscle area
	Bioimpedance analysis: whole body fat-free mass index
Physical and psychosocial functioning	EORTC QLQ-C30
	ECOG questionnaire
	Karnofsky performance score
	Electric activity meters
	Checklists of specific activities

CRP, C-reactive protein; CT, computed tomography; MRI, magnetic resonance imaging; DEXA, dual-energy X-ray absorptiometry; EORTC QLQ-C30, European Organization for Research and Treatment of Cancer quality of life questionnaire C-30; ECOG, Eastern Cooperative Oncology Group.

Evaluation of food intake should be routinely performed. At the minimum, patients can be asked to estimate their overall food intake in relation to normal intake with dietary or recall records. Another simple method for prospective third-party assessment of food intake is the percentile calculation of food consumed at each meal by a family member (Bruera and Sweeney, 2000). Patients should also be evaluated for underlying factors that contribute to reduced food intake, such as lack of appetite, chemosensory disturbances, dysphagia, decreased gastrointestinal (GI) motility, pain, and fatigue.

A key component of pancreatic cancer cachexia is hypercatabolism due to direct tumor metabolism, systemic inflammation, or other tumor-mediated effects. Hypercatabolism due to systemic inflammation can be assessed using serum CRP levels (Moses et al., 2009). Indirect indices of catabolism include responsiveness to chemotherapy and rate of disease progression.

Cancer cachexia is characterized by ongoing skeletal muscle loss. There are various methods for muscle mass assessment: cross-sectional imaging with computed tomography (CT) or magnetic resonance imaging (MRI); appendicular skeletal muscle index obtained from DEXA; mid-upper-arm muscle area by anthropometry; and whole body fat-free mass index determined by bioimpedance analysis (Simons et al., 1995; Prado et al., 2009; Fearon et al., 2011; Di Sebastiano and Mourtzakis, 2012). Imaging-based methods of muscle mass assessment can quantify changes in body composition, including skeletal muscle wasting, altered distribution of body fat, and pathological accumulation of lipids in various tissues. MRI can measure the volume of the quadriceps muscle with a coefficient of variation < 1%. Diagnostic CT scans can be used to estimate abdominal muscle cross-sectional area at the L3 level, which can be extrapolated to whole body lean body mass. These modalities are usually reserved for research purposes and not routinely used in the clinic.

A comprehensive approach, including history, physical examination, and various imaging studies can aid in recognizing the phenomenon termed sarcopenic obesity or cachexia hidden in the context of obesity. Even at the time of diagnosis, approximately 40% of overweight or obese pancreatic cancer patients have substantial ongoing skeletal muscle wasting (Tan et al., 2009). Early detection of sarcopenic obesity is important because it has been shown to be an independent prognostic factor for decreased survival in pancreatic cancer patients (Tan et al., 2009).

Cancer cachexia can have a profound adverse effect on physical and psychosocial functioning. Patients report altered body images, which can significantly impact emotions and relationships. The method of choice for evaluating functional effects of cancer cachexia is the routine use of patient-reported physical functioning. This assessment can be obtained using the European Organization for Research and Treatment of Cancer (EORTC) Quality of Life Questionnaire (QLQ)-C30 or the Eastern Cooperative Oncology Group (ECOG) questionnaire. Physician-reported physical activity (Karnofsky performance score) and objective methodologies such as electric activity meters or checklists of specific activities can also be used to assess physical functioning. A recent study with subjects wearing an electric activity monitor showed that the level of physical activity in cachectic cancer patients is reduced by about 40% (Dahele et al., 2007).

## Mechanisms of cancer cachexia

**Figure 1:**
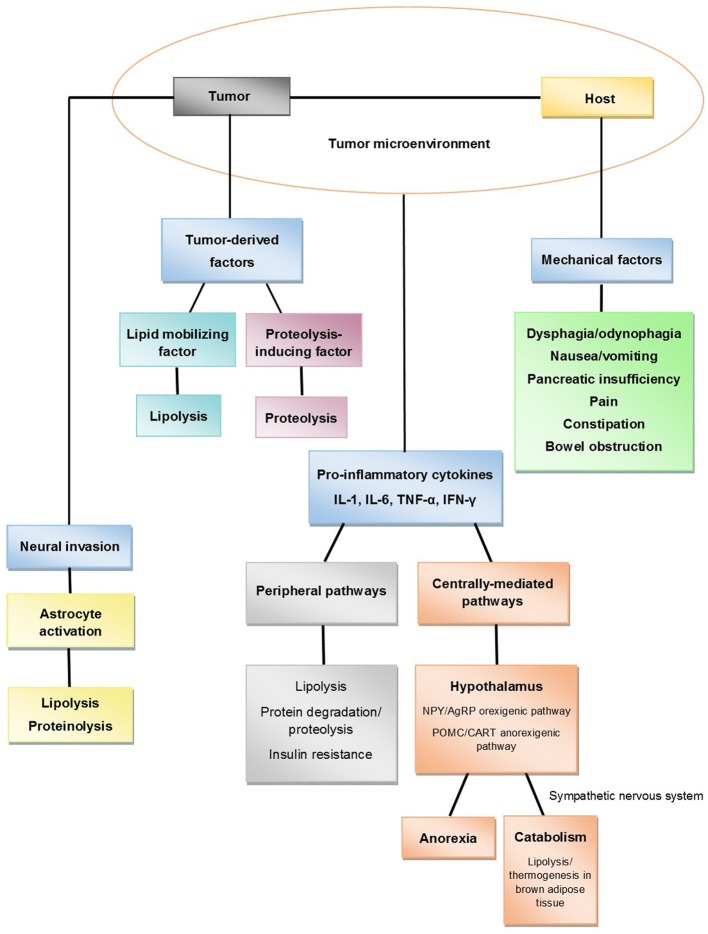
**Proposed mechanisms of pancreatic cancer cachexia**.

The pathophysiology of cancer cachexia is characterized by negative protein and energy balance driven by a combination of reduced food intake and increased metabolism (Fearon, 2008, 2012; Fearon et al., 2011). This process involves complex interactions between the host and the tumor (Figure 1). There are mechanical factors that contribute to reduced food intake. There is evidence that anorexia and hypercatabolism are driven by cytokines, circulating hormones, neuropeptides, neurotransmitters, and tumor-derived factors. In addition, recent studies have discovered other potentially significant processes involved in pancreatic cancer cachexia, including neural invasion and abnormalities in the muscle microenvironment. This section will review current proposed mechanisms that lead to the development of this disease process.

### Mechanical factors

Reduced food intake can promote and maintain cancer-associated weight loss (Wigmore et al., 1997b). Mechanical digestive abnormalities can result in a lack of appetite and reduced food intake. Patients with pancreatic cancer suffer from pain, fatigue, nausea, dysphagia, gastroparesis, duodenal stenosis, pancreatic insufficiency and malabsorption, and constipation (Deutsch and Kolhouse, 2004). These symptoms are the direct consequence of tumor invasion, which can result in the obstruction of the pancreatic duct and/or GI tract, particularly the second portion of the duodenum. Many patients will undergo pancreaticoduodenectomy for the resection of a pancreatic head mass. This procedure can worsen pancreatic insufficiency and decrease oral intake.

### Cytokines and systemic inflammation

Systemic inflammation plays an important role in the pathophysiology of pancreatic cancer cachexia. Elevated CRP levels (CRP > 10 mg/L), an indirect measure of systemic inflammation, has been associated with cachexia and poor prognosis in pancreatic cancer patients (Fearon et al., 2006b). Elevated cytokine levels, including IL-6 and IL-10, have been associated with poor performance, weight loss, and decreased survival in pancreatic cancer patients (Ebrahimi et al., 2004; Moses et al., 2009). Recent evidence strongly suggest that cytokines produced by tumor cells or released by the host as a response to the cancer affect various pathways that lead to anorexia and a hypercatabolic state (Figure 1). These pathways can be divided into central pathways, which are hypothalamus-mediated, and peripheral pathways, which involve direct lipolysis and proteolysis.

#### Centrally-mediated pathways

Under normal conditions, energy homeostasis is a highly regulated system of controls. The hypothalamus controls energy intake by integrating peripheral signals that convey information on energy and adiposity status. These inputs are transduced into neuronal responses and, via signaling pathways, behavioral responses. Current evidence suggests that systemic inflammation plays a critical role in inducing cancer anorexia by triggering a complex neurochemical cascade (Laviano et al., 2003; Fearon et al., 2013; Suzuki et al., 2013; Tuca et al., 2013). Increased cytokine expression from tumor growth prevents the hypothalamus from responding appropriately to peripheral signals by persistent stimulation of anorexigenic pathways and inhibition of orexigenic pathways (Suzuki et al., 2013; Tuca et al., 2013).

Cancer anorexia may be partially due to derangement of peripheral signaling transduction into neuronal responses by the hypothalamus. There are two pathways that control energy expenditure and food intake within the hypothalamus: neuropeptide Y (NPY)/Agouti-related peptide (AgRP) neurons that stimulate energy intake and pro-opiomelanocortin (POMC)/cocaine and amphetamine-regulated transcript (CART) neurons that inhibit intake. Some studies suggest that cancer cachexia is associated with hyperactivation of the POMC/CART pathway which may be triggered by IL-1 and other pro-inflammatory cytokines (Wisse et al., 2001; Marks and Cone, 2003; Marks et al., 2003; Scarlett et al., 2007).

Leptin is a protein involved in regulating energy intake and expenditure. Leptin reduces appetite and increases energy expenditure via the central nervous system (CNS). Through feedback signaling, leptin controls the production, and activation of hypothalamic neuropeptides that regulate food intake and energy expenditure, including NPY and corticotropin-releasing factor (CRF). Since leptin is primarily released by adipose tissue, decreased body fat mass or starvation leads to a decrease in leptin levels. Low leptin levels allow for increased production, release, and action of NPY, a potent orexigenic peptide, which subsequently results in the activation of the NPY/AgRP pathway. Additionally, low leptin levels result in decreased activity of anorexigenic neuropepetides, such as CRF and melanocortin. Studies have demonstrated that cytokines, such as tumor necrosis factor-alpha (TNF-α) and interleukin1 (IL-1) increase the expression of leptin mRNA in adipocytes and plasma levels of leptin despite starvation (Grunfeld et al., 1996; Janik et al., 1997; Sarraf et al., 1997; Finck et al., 1998). Therefore, an increased leptin level may contribute to cancer anorexia by preventing the normal compensatory mechanisms that should occur with decreased food intake. However, other studies have shown that these cytokines can induce anorexia even in the absence of leptin (Faggioni et al., 1997). Animal and clinical studies have also demonstrated that leptin levels are not elevated in tumor-bearing rats and patients with cancer cachexia (Simons et al., 1997; Wallace et al., 1998; Mantovani et al., 2000; Bing et al., 2001). Recent evidence suggests that in cancer cachexia IL-1 and TNF-α mimic leptin signaling and interfere with the orexigenic response to reduced leptin levels (Inui, 1999; Suzuki et al., 2013). Therefore, even with decreased adiposity, there continues to be suppression of the orexigenic response and stimulation of the anorexigenic pathway, resulting in unopposed anorexia and increased energy expenditure.

Serotonin may also play an important role in the development of cancer anorexia through the melanocortin system. Studies have established that IL-1 stimulates the release of hypothalamic serotonin (Shintani et al., 1993). Elevated serotonin levels, in turn, contribute to the persistent activation of POMC/CART neurons, resulting in decreased appetite and anorexia (Heisler et al., 2002). Studies have demonstrated elevated plasma and cerebrospinal fluid concentrations of tryptophan, a precursor of serotonin, in patients with evidence of cancer cachexia compared to healthy controls or cancer patients without cachexia (Cangiano et al., 1990). Plasma tryptophan concentration normalized, and food intake improved after tumor removal (Cangiano et al., 1994). These findings suggest that hypothalamic serotonin may be an important factor in the pathogenesis of cancer cachexia and a potential therapeutic target.

These hypothalamic pathways and neuropeptides also have catabolic effects. The POMC/CART anorexigenic pathway increases the sympathetic nervous system activity, which causes induction of mitochondrial uncoupling proteins, such as UCP-1 and UCP-2 (Li et al., 2002; Arruda et al., 2010). UCP-1 channels protons across the inner mitochondrial membrane without ATP production, resulting in thermogenesis and energy expenditure in brown adipose tissue (Li et al., 2002; Arruda et al., 2010).

#### Peripheral pathways

Cytokines not only corroborate and sustain the neurochemical changes responsible for anorexia, they have also been shown to induce lipolysis, muscle catabolism, and the hepatic acute phase protein response (APPR) through various pathways. These processes lead to the development of uncompensated loss of muscle and adipose tissue mass.

***TNF-α***. TNF-α was first identified as a cachexia-inducing factor in chronic diseases. It may have properties that promote lipolysis, impair lipogenesis, and induce muscle degradation. It has been shown to induce lipolysis *in vitro* with increases in glycerol release in mouse and human adipocytes, likely through downregulation of perilipin expression (Rydén et al., 2004). Perilipin coats intracellular lipid droplets and acts as a barrier to lipolysis. Decreased perilipin expression subsequently enables hormone-sensitive lipase (HSL), a key regulator of lipolysis, to access the surface of lipid droplets for breakdown (Zhang et al., 2002; Rydén et al., 2004). TNF-α also has an inhibitory effect on adipocyte differentiation, resulting in impaired lipogenesis (Cawthorn et al., 2007; Hammarstedt et al., 2007).

Animal studies also suggest that TNF-α is involved in muscle loss in cancer cachexia. Mouse models have shown that TNF-α may induce muscle protein degradation through formation of reactive oxygen species (ROS). Oxidative stress results in the activation of nuclear factor κB (NFκB) which, in turn, activates the ubiquitin-proteasome pathway (Llovera et al., 1998; Li and Reid, 2000). Moreover, TNF-α has been shown to increase expression of the 1.2- and 2.4-kb transcripts of ubiquitin and the ubiquitin ligase atrogin 1/MAFbx in skeletal muscle (Llovera et al., 1998; Li and Reid, 2000). In addition to protein degradation, TNF-α has been shown to inhibit myogenesis *in vitro* through NFκB-mediated downregulation of MyoD transcripts (Guttridge et al., 2000).

Although these findings suggest a role for TNF-α in lipolysis and proteolysis, its importance in cancer cachexia is an active area of debate. Results from studies measuring levels of TNF-α in patients with cancer cachexia have been conflicting. Some studies have shown detectable levels of TNF-α in the serum of pancreatic cancer patients with TNF-α levels inversely correlating with body weight and BMI; other studies involving patients with advanced cancers have shown no correlation between circulating TNF-α levels, weight loss, and anorexia (Maltoni et al., 1997; Karayiannakis et al., 2001; Rydén et al., 2008). Therefore, the origin and relevance of TNF-α to cancer cachexia remains unclear.

***IL-6.*** IL-6 is another important cytokine in pancreatic cancer cachexia. IL-6 secretion is induced by TNF-α; it acts synergistically with TNF-α in many of its actions including stimulation of other cytokines. Circulating levels of IL-6 correlate with weight loss and reduced survival in pancreatic cancer patients (Ebrahimi et al., 2004; Martignoni et al., 2005; Moses et al., 2009). Although the role of IL-6 in lipolysis is not well established, a recent study has shown enhanced IL-6 signaling in brown adipose tissue in cachectic tumor-bearing mice suggesting that it may play a direct role in the activation of thermogenesis (Tsoli et al., 2012). More importantly, IL-6 is known to activate the hepatic APPR and trigger tissue catabolism. The murine C-26 cachexia model has shown that increasing levels of IL-6 correlated with the development of cachexia; treatment with an IL-6 neutralizing antibody attenuated the development of weight loss (Strassmann et al., 1992). Moses et al. found that pancreatic cancer patients with cachexia had elevated CRP levels and stimulated IL-6 production (Moses et al., 2009). Although various cytokines and hormones affect hepatocyte protein metabolism, IL-6 is known as the principal regulator of APPR in human hepatocytes (Castell et al., 1990). There is a strong correlation between increased peripheral blood mononuclear cells (PBMC) production of IL-6 and the presence of elevated APPR (Martignoni et al., 2005, 2009; Moses et al., 2009). The activation of hepatic APPR subsequently results in hypercatabolism through reprioritization of body protein metabolism from skeletal muscle to production of acute phase proteins (Fearon et al., 1999). There appears to be a two- to three-fold increase in fibrinogen production and increase in serum CRP levels (Preston et al., 1998). Production of these acute phase proteins by the liver is associated with mobilization of peripheral amino acid stores primarily from skeletal muscle contributing to the loss of lean tissue and catabolism. Overproduction of IL-6 and elevated APPR have been associated with decreased survival in patients with pancreatic cancer cachexia (Moses et al., 2009).

### Tumor-derived factors

In addition to cytokines and systemic inflammation, tumor-derived factors contribute to metabolic abnormalities that give rise to pancreatic cancer cachexia. Two of the most well studied factors are lipid mobilizing factor (LMF) and proteolysis-inducing factor (PIF). The presence and role of other factors that contribute to pancreatic cancer cachexia are currently being investigated.

#### Lipid mobilizing factor

Todorov et al. isolated a LMF from a cachexia-inducing murine tumor (MAC16 adenocarcinoma) model and the urine of patients with unresectable pancreatic cancer and weight loss (Todorov et al., 1998). The material was 43 kDA and was found to be homologous with the plasma protein zinc-α2-glycoprotein (ZAG) (Todorov et al., 1998). Pancreatic cancer patients with weight loss generally had LMF/ZAG in the urine, but it was absent from patients without weight loss or normal subjects (Todorov et al., 1998). A recent study identifying serum proteins involved in pancreatic cancer cachexia identified LMF/ZAG as a possible marker (Felix et al., 2011). Moreover, immunohistochemical analysis demonstrated LMF/ZAG expression in pancreatic cancer cells and in the peritumoral stroma (Felix et al., 2011). Patients with cachexia had stronger immunostaining compared to pancreatic cancer patients without cachexia or normal subjects (Felix et al., 2011).

*In vivo* studies have shown that LMF/ZAG causes selective loss of carcass fat without change in body water or nonfat mass (Hirai et al., 1998). LMF/ZAG directly induces lipolysis by stimulating adenylate cyclase in a GTP-dependent process; this process is postulated to be mediated by β3 adrenergic receptors (Hirai et al., 1998; Khan and Tisdale, 1999; Russell et al., 2002). Hirai et al. showed an increase in serum levels of glycerol and 3-hydroxybutyrate after treating mice with LMF/ZAG. They also showed a significant increase in oxygen uptake by brown adipose tissue suggesting that LMF/ZAG promotes lipid utilization (Hirai et al., 1998). In addition, LMF/ZAG has been shown to increase lipid oxidation using the production of ^14^CO_2_ from [^14^C-carboxy]triolein (Russell and Tisdale, 2002). This function is achieved by directly activating the expression of mitochondrial UCPs. LMF/ZAG induces increased expression of UCP-1, UCP-2, and UCP-3 in brown adipose tissue, and UCP-2 in skeletal muscle and liver (Bing et al., 2002). The effect of LMF/ZAG on lipid oxidation and utilization is also likely mediated by β3 adrenergic receptors. LMF/ZAG also increases the sensitivity of white adipose tissues to the lipolytic effects of other stimuli, including catecholamines (Islam-Ali et al., 2001). Adipocyte plasma membranes have Gs α-subunits and Gi α-subunits, which stimulate and inhibit adenylate cyclase, respectively. LMF/ZAG increases Gαs expression and decreases Gαi expression, which favor mobilization of lipid stores from adipocytes and facilitate a catabolic state (Islam-Ali et al., 2001). LMF/ZAG not only increases lipid mobilization through various pathways but it also increases substrate utilization and activates mitochondrial oxidative pathways in brown adipose tissue resulting in lipolysis, increased energy expenditure, and hypercatabolism.

### Proteolysis-inducing factor

PIF was discovered in 1996 using a MAC16 adenocarcinoma mouse model of cachexia. Todorov et al. reported the discovery of a glycoprotein of molecular mass 24 kDa that produced cachexia *in vivo* by inducing skeletal muscle catabolism (Todorov et al., 1996). The same material was isolated from urine of cachectic cancer patients, but not from patients with weight loss due to trauma, cancer patients with little or no weight loss, and normal subjects. PIF was detected in the urine of 80% of pancreatic cancer patients with significantly greater total weight loss and rate of weight loss than patients who did not have PIF in their urine (Wigmore et al., 2000b). Immunochemistry demonstrated that the 24 kDa material is present in the cytoplasm of GI tumors, including pancreatic adenocarcinoma (Cabal-Manzano et al., 2001). Enzymatic degradation of PIF suggests that it consists of a peptide core with molecular weight 4000 Da that is extensively *N*- and *O*-glycosylated to give a total molecular mass of 24 kDa (Todorov et al., 1997). Examination of the sequence of the human genome revealed the gene for the polypeptide core of PIF is located in chromosome 12; two proteins, dermicidin and Y-P30, have been reported to have 100% homology (Schittek et al., 2001; Cunningham et al., 2002). However, enzymatic degradation has shown that the oligosaccharide chains are essential for the biologic activity of PIF (Todorov et al., 1997).

When administered intravenously to normal mice, PIF isolated from urine of cancer cachexia patients induced cachexia without reduction in food and water intake (Cariuk et al., 1997). Analysis of body composition demonstrated that the majority of weight loss involved loss of lean body mass (Cariuk et al., 1997). This decrease in lean body mass had two components: an increase in protein degradation by 50% and a decrease in protein synthesis by 50% observed in gastrocnemius muscle (Lorite et al., 1997). Some studies suggest that PIF-mediated protein degradation may involve the ubiquitin-proteasome proteolytic pathway. Administration of PIF to normal mice caused an increase in mRNA levels for ubiquitin, E2_14*k*_ and the C9 proteasome subunit. Therefore, protein degradation likely occurs through increased expression of the ubiquitin-proteosome pathway in skeletal muscle; this process is thought to be mediated by the activation of NFκB (Lorite et al., 2001; Whitehouse and Tisdale, 2003; Wyke and Tisdale, 2005). PIF has also been shown to induce total protein degradation and myosin depletion while actin levels remain unchanged (Wyke and Tisdale, 2005).

The mechanism for NFκB activation by PIF is not fully understood. It does involve release of arachidonic acid from membrane phospholipids with rapid metabolism to eicosanoids by phospholipase A_2_ (PLA_2_) (Smith and Tisdale, 2003). PIF has been shown to increase expression of PLA_2_ (Smith and Tisdale, 2003). One of the eicosanoids formed in response to PIF, 15-hydroxyeicosatetraenoic acid (15-HETE), can induce muscle degradation in murine muscle cells (Wyke et al., 2005). 15-HETE may be involved in the activation of protein kinase C (PKC), which is important in the activation of NADPH oxidase (Whitehouse et al., 2003; Smith et al., 2004; Wyke et al., 2005). Activation of NADPH oxidase and generation of ROS play a key role in PIF-induced expression of the ubiquitin-proteasome pathway leading to muscle degradation (Smith et al., 2004; Russell et al., 2007). Increased ROS activates Iκ B kinase (IKK) which leads to phosphorylation and degradation of Iκ B; this process, in turn, releases NFκB from its inactive cytosolic complex (Smith et al., 2004).

PIF not only results in protein degradation, it also causes inhibition of protein synthesis. PIF induces the activation/phosphorylation of double-stranded RNA-dependent protein kinase (PKR) (Eley and Tisdale, 2007). The activation of PKR leads to the phosphorylation of eIF2, which inhibits translation initiation and protein synthesis (Eley and Tisdale, 2007). In addition, PKR is known to activate IKK resulting in the nuclear accumulation of NFκB and increased expression and activity of the ubiquitin-proteasome pathway (Zamanian-Daryoush et al., 2000).

In addition to its direct effects on skeletal muscles, PIF may play a role in increasing hepatic cytokine production. Treatment of cultures of human hepatocytes with PIF resulted in activation of NFκB and signal transducers and activators of transcription (STAT3), which caused an increased production of IL-6, IL-8, and CRP, as well as decreased production of transferrin (Watchorn et al., 2001). A similar effect was observed in human Kupffer cells and monocytes and resulted in increased production of TNF-α, IL-6, and IL-8 (Watchorn et al., 2005). PIF may likely contribute to APPR seen in pancreatic cancer cachexia.

### Other proposed mechanisms

#### Pax7 dysregulation

A recent study provides evidence for a different pathway involved in pancreatic cancer related muscle wasting. Pax7 is a self-renewing transcription factor expressed in various muscle cells, including satellite cells and other myogenic progenitor cells. He et al. demonstrated that NFκB activation in satellite cells resulted in the dysregulation of Pax7, which suppressed expression of MyoD and myogenin (Olguin and Olwin, 2004; He et al., 2013). This process subsequently blocked myogenic differentiation and inhibited myoblast fusion leading to impaired regeneration and muscle wasting (He et al., 2013). They also demonstrated that Pax7 was induced by serum factors from cachectic mice and pancreatic cancer patients in an NFκB-dependent manner both *in vitro* and *in vivo* (He et al., 2013). However, it remains unclear what circulating factors lead to NFκB activation and Pax7 dysregulation.

#### Neural invasion

Recent studies have shown that neural invasion, which commonly occurs in pancreatic cancer, is related to cachexia and astrocyte activation in pancreatic cancer patients (Mitsunaga et al., 2008; Imoto et al., 2012). Nerve damage from intraneural tumors of pancreatic cancer can activate astrocytes and microglia in the spinal cord. These activated astrocytes subsequently induce lipolysis and muscle atrophy, although the mechanisms leading to cachexia require further investigation (Imoto et al., 2012). These activated astrocytes may increase sympathetic nervous system activity, which is known to cause lipolysis in adipose tissue and muscle atrophy (Li et al., 2002).

### Management of cancer cachexia

Clinical management of cachexia is currently limited and complex. Best supportive care practices are important in managing secondary causes of anorexia including pain, nausea, pancreatic insufficiency, and constipation. In addition, current treatment strategies are based on the following factors: oncological therapy with optimal control of the tumor; nutritional support; and pharmacological treatment. Since cancer cachexia is a multifactorial syndrome, successful treatment will likely involve a multimodal approach.

### Nutritional support

Nutritional risk is highest among pancreatic cancer patients (Bozzetti and Group, 2009). Early involvement of dieticians and nutrition assessment programs are essential to guide management. Nutritional support is an integral part of pancreatic cancer cachexia management and involves providing dietary advice, oral nutritional supplementation, enteral nutrition, and parenteral nutrition (Ottery, 1996; Nitenberg and Raynard, 2000; Jatoi and Loprinzi, 2001; el-Kamar et al., 2003).

Dietary recommendations can significantly increase oral caloric and protein intake (Ovesen et al., 1993). Several studies evaluating the role of oral nutritional supplementation among patients with pancreatic cancer demonstrated improvement in weight and appetite (Fearon et al., 2003; Bauer and Capra, 2005). Oral supplementation with compounds such as L-Carnitine and omega-3 fatty acids may have benefits as well (Barber et al., 1999; Kraft et al., 2012). A small multicenter randomized double-blind trial demonstrated a significant improvement in weight and body mass composition as well as quality of life with L-Carnitine supplementation in patients with advanced pancreatic cancer (Kraft et al., 2012).

In patients with swallowing difficulties or severe dysphagia, a complete enteral diet can be administered using a nasogastric tube or gastrostomy tube. Enteral feeding can be associated with significant morbidity due to aspiration, pneumonia, and diarrhea. In a select group of patients with bowel dysfunction and progressive weight loss despite enteral support, parenteral nutrition may provide a temporary benefit or stabilization in nutritional status (Pelzer et al., 2010).

Artificial nutrition can limit nutritional deterioration in cachectic cancer patients and improve certain metabolic and nutritional indices. However, the nutritional response is typically limited. It is also lower than responses observed in malnourished non-cancer patients receiving equivalent artificial nutrition (Nixon et al., 1981). Patients with pancreatic cancer cachexia require a multimodal approach to disease management (DeWys, 1979).

### Pharmacological approach

Various drugs have been studied in the treatment of cancer cachexia. Their mechanisms of action are based on modulation of cytokines, hormones, or other pathways involved in the pathophysiology of cancer cachexia. Table 3 summarizes drugs and their pharmacologic activity with proven or potential effects on pancreatic cancer cachexia.

**Table 3:** **Pharmacological approach to pancreatic cancer cachexia**.

**Drugs**	**Mechanism of action**	**References**	**Level of evidence**
Progestogens (megestrol acetate and medroxyprogesterone acetate)	Appetite stimulation Decrease production and release of cytokines (IL-1, IL-6, TNF-α) Stimulation of NPY Decrease production of serotonin in PBMC	Bruera et al., 1990; Loprinzi et al., 1990, 1993a,b; McCarthy et al., 1994; Mantovani et al., 1998a,b; Westman et al., 1999; Deutsch and Kolhouse, 2004; Pascual López et al., 2004; Leśniak et al., 2008	I
Corticosteroids (prednisone, dexamethasone, methylprednisolone)	Appetite stimulation Not well understood Likely from inhibition of IL-1, TNF-α, and leptin Stimulation of NPY	Willox et al., 1984; Bruera et al., 1985; Plata-Salamán, 1991; Loprinzi et al., 1999;	I
Cannabinoids (dronabinol)	Appetite stimulation, anti-emetic Interaction with endorphin receptors Interference with IL-1 synthesis Activation of cannabinoid receptor involved in the neurochemical circuit of leptin Inhibition of prostaglandin synthesis	Nelson et al., 1994; Jatoi et al., 2002	II
NSAIDs (COX-2 inhibitors, indomethacin, ibuprofen)	Anti-inflammatory Decrease production and release of acute phase proteins and pro-inflammatory cytokines Inhibit prostaglandin synthesis	Gelin et al., 1991a,b; Lundholm et al., 1994; McMillan et al., 1995, 1997, 1999; Preston et al., 1995; Wigmore et al., 1995; Lai et al., 2008	II
Thalidomide	Anti-inflammatory and immunomodulatory properties Downregulate production of TNF-α and other cytokines Inhibit NFκB Downregulate COX-2 Inhibit angiogenesis	Sampaio et al., 1991; Bruera et al., 1999; Gordon et al., 2005	II
Omega-3 fatty acids (eicosapentaenoic acid)	Anti-inflammatory and immunomodulatory properties Decrease production of cytokines (IL-1, IL-6, TNF-α) Inhibit downstream effects of LMF and PIF	Tisdale and Beck, 1991; Meydani et al., 1993; Tisdale, 1996; Wigmore et al., 1996, 1997a, 2000b; Barber et al., 1999; Hussey and Tisdale, 1999; Jatoi et al., 2004; Fearon et al., 2006a	Conflicting

NSAIDs, non-steroidal anti-inflammatory drugs; NPY, neuropeptide Y; IL-1, interleukin-1; IL-6, interleukin-6; TNF-α, tumor necrosis factor-alpha; PBMC, peripheral blood mononuclear cells; NFκB, nuclear factor κ B; COX-2, cyclooxygenase-2; LMF, lipid mobilizing factor; PIF, proteolysis-inducing factor.

#### Progestogens

Megestrol acetate is a semi-synthetic progesterone currently used as an appetite stimulant. When megestrol acetate was first introduced in the treatment of disseminated breast and endometrial cancer, patients developed weight gain and increased appetite as a side effect. Multiple trials demonstrated that megestrol acetate (480–800 mg/day) resulted in significant improvement in appetite, food intake, nausea, and weight gain among patients with cancer cachexia, including those with pancreatic cancer (Bruera et al., 1990; Loprinzi et al., 1990, 1993a; Westman et al., 1999; Deutsch and Kolhouse, 2004). In 1993, the Food and Drug Administration (FDA) approved megestrol acetate for the treatment of cancer anorexia-cachexia syndrome as well as cachexia due to chronic conditions, including human acquired immunodeficiency syndrome (AIDS). Megestrol acetate is typically well-tolerated with low incidence of adverse effects, such as rash, adrenal insufficiency, hyperglycemia, and thromboembolic events. The increase in thromboembolism has an incidence of less than 5% (Loprinzi et al., 1990). Since its approval, various meta-analyses have confirmed that megestrol acetate increases appetite, weight, and quality of life compared to placebo or other drugs potentially active in the management of cancer cachexia (cisapride, dronabinol, corticosteroids, nandrolone) (Pascual López et al., 2004; Leśniak et al., 2008). The efficacy of megestrol acetate appears to be dose-dependent (Loprinzi et al., 1993a). Based on body composition analysis, megestrol acetate causes weight gain predominantly from an increase in adipose tissue and less from an increase in body fluid (Loprinzi et al., 1993b). There was no improvement in survival demonstrated in patients treated with megestrol acetate (Westman et al., 1999; Leśniak et al., 2008).

The pharmacologic activity of megestrol acetate in appetite stimulation and weight gain may be related to decreased production and release of pro-inflammatory cytokines (IL-1, IL-6, TNF-α) and stimulation of NPY in the hypothalamus (McCarthy et al., 1994; Mantovani et al., 1998a,b). Another progestogen, medroxyprogesterone acetate, was shown to decrease *in vitro* production of cytokines and serotonin by PBMC of cancer patients (Mantovani et al., 1998a,b).

#### Corticosteroids

Corticosteroids are effective in inducing an increase in appetite, food intake, weight gain, and sense of well-being (Willox et al., 1984; Bruera et al., 1985; Loprinzi et al., 1999). However, the effects are short lived (less than 4 weeks) and associated with long-term side effects, such as insulin resistance, fluid retention, steroid-induced myopathy, skin fragility, adrenal insufficiency, and sleep and cognitive disorders (Loprinzi et al., 1999). The mechanism of action in cancer cachexia is not well understood but is likely related to the inhibition of IL-1, TNF-α, and leptin as well as the stimulation of NPY (Plata-Salamán, 1991). Because of their short term symptomatic benefits but long term adverse effects, corticosteroids may be useful in patients with short expected survival.

#### Cannabinoids

Dronabinol is effective in reducing nausea and increasing appetite with associated weight stabilization. A phase II trial showed that dronabinol reduced anorexia in 68% of patients, but 16% of patients had to suspend treatment due to toxicity (Nelson et al., 1994). Dronabinol has many adverse effects on the CNS. The main side effects include euphoria, hallucinations, psychosis, vertigo, and cardiovascular disorders. Appetite stimulation appears to be mediated by interaction with endorphin receptors, interference with IL-1 synthesis, activation of cannabinoid receptors involved in the neurochemical circuit of leptin, and prostaglandin synthesis inhibition.

A controlled clinical trial by Jatoi et al. compared megestrol acetate and dronabinol in patients with cancer cachexia (Jatoi et al., 2002). A total of 469 patients were treated with megestrol acetate 800 mg/day or dronabinol 2.5 mg/12 h or both. There was a greater increase in appetite and weight in the megestrol acetate group compared to the dronabinol group: 75 vs. 49% (*P* = 0.0001) for appetite, respectively and 11 vs. 3% (*P* = 0.02) for weight gain of at least 10% from baseline, respectively (Jatoi et al., 2002). The combination treatment group resulted in no significant differences in appetite and weight when compared to the megestrol acetate only group (66 vs. 75%, *P* = 0.17, for appetite and 8 vs. 11%, *P* = 0.43, for =10% weight gain, respectively). Megestrol acetate appears to be superior to dronabinol although the cannabinoid is still able to trigger an increase in appetite and reduction in nausea. It serves as an alternative option as an appetite stimulant and anti-emetic.

#### Anti-inflammatory agents

Systemic inflammation is an important contributor to the pathophysiology of pancreatic cancer cachexia. Pro-inflammatory cytokines, such as TNF-α, IL-1, and IL-6, have been implicated in the development of cancer cachexia and have been shown to exhibit synergistic effects. Therefore, multiple therapeutic strategies have been developed to curtail the inflammatory response by blocking the synthesis or action of cytokines.

Non-steroidal anti-inflammatory drugs (NSAIDs), including cyclooxygenase-2 (COX-2) inhibitors, indomethacin, and ibuprofen, reduce release of acute phase proteins and cytokines (McMillan et al., 1995; Preston et al., 1995; Wigmore et al., 1995). In animal studies, inhibition of prostaglandin synthesis attenuated tumor progression and decreased incidence of cancer cachexia (Gelin et al., 1991a,b). One possible explanation is that cytokines depend on signal transduction mediated by eicosanoids; NSAIDs inhibit prostaglandin synthesis and thereby block downstream effects of systemic inflammation. Lundholm et al. demonstrated that indomethacin use may prolong survival in cachectic patients with metastatic solid tumors, including pancreatic cancer (Lundholm et al., 1994). Other controlled clinical trials have shown that ibuprofen can decrease CRP levels, increase body weight and muscle mass, and improve quality of life, especially when combined with progestogens (Wigmore et al., 1995; McMillan et al., 1997; Lai et al., 2008). McMillan et al. recruited 73 patients with advanced GI cancers, predominantly pancreatic cancer (67% of patients), and cancer cachexia (McMillan et al., 1999). This multicenter randomized controlled trial demonstrated that taking ibuprofen (1200 mg/day) combined with megestrol acetate (480 mg/day) resulted in a significant increase in weight and improved quality of life compared to patients taking megestrol acetate alone (McMillan et al., 1999). Observed side effects were similar in both groups including venous thrombosis, upper GI bleeding, and ascites. However, due to disease progression, the attrition rate was quite high with 63% of patients failing to complete the 12-week assessment. These preliminary results are promising, but further larger studies are needed to evaluate the clinical role of NSAIDs in the management of pancreatic cancer cachexia.

Thalidomide is known to have anti-inflammatory and immunomodulatory properties. It has been shown to downregulate the production of TNF-α and other cytokines, inhibit NFκB, downregulate COX-2, and inhibit angiogenesis (Sampaio et al., 1991). Multiple small studies have demonstrated the efficacy of thalidomide in improving appetite, weight gain, and sensation of well-being (Bruera et al., 1999; Gordon et al., 2005). Gordon et al. reports a single-center double-blind placebo-controlled randomized clinical trial of thalidomide in 50 pancreatic cancer patients with cachexia. Patients were randomized to take thalidomide 200 mg/day or placebo. Patients in the thalidomide group compared to the placebo group had a significant improvement in weight (0.37 vs. −2.21 kg, *P* = 0.005) and lean body mass (1.0 cm^3^ in arm muscle mass vs. −4.46 cm^3^, *P* = 0.002) after 4 weeks (Gordon et al., 2005). Thalidomide was overall well-tolerated. Adverse reactions included peripheral neuropathy, dizziness, somnolence, constipation, rash, and possible increased risk of venous thromboembolism in the setting of malignancy. These initial results are positive but further clinical trials are needed to confirm the efficacy of thalidomide in treating pancreatic cancer cachexia.

The omega-3 fatty acids, eicosapentaenoic acid (EPA) and docosahexaenoic acid (DHA), are known to have immunomodulatory properties and have been shown to suppress production of pro-inflammatory cytokines, including IL-1, TNF-α, and IL-6 by PBMC (Meydani et al., 1993; Wigmore et al., 1997a). EPA may also inhibit the downstream effects of LMF and PIF (Tisdale and Beck, 1991; Tisdale, 1996; Hussey and Tisdale, 1999). Early studies associated fish oil supplementation containing both EPA and DHA as well as high-purity EPA administration with weight stabilization in patients with unresectable pancreatic cancer (Wigmore et al., 1996, 2000b). A small pilot study also showed that the use of EPA with oral nutritional supplements resulted in significant increase in weight, dietary intake, and performance status in cachectic patients with advanced pancreatic cancer (Barber et al., 1999). However, recent data from a multicenter double-blind placebo-controlled randomized clinical trial suggest that single agent EPA administration is not effective in treating cancer cachexia (Fearon et al., 2006a). Another multicenter clinical trial comparing the effects of EPA supplement, megestrol acetate, and combination treatment found that megestrol acetate alone is more effective than EPA in increasing weight (Jatoi et al., 2004). EPA was comparable to megestrol acetate with respect to appetite gain, survival, and quality of life (Jatoi et al., 2004). Combination therapy did not have additional benefits to megestrol acetate alone (Jatoi et al., 2004). The role of EPA in cancer cachexia management remains uncertain although recent data suggest that EPA supplementation may not be effective as a single agent or even in combination regimens in the management of pancreatic cancer cachexia.

Pancreatic cancer cachexia is a complex multifactorial syndrome. Successful management may require a multimodal approach with nutritional supplementation and pharmacological treatment. Recent data from a large multicenter trial suggest that combination therapy with megestrol acetate (320 mg/day), EPA supplementation, L-carnitine (4 g/day), and thalidomide (200 mg/day) is significantly more effective in improving lean body mass and appetite than single agents (Mantovani et al., 2010). Combination pharmacological therapy with nutritional supplementation in the context of best supportive care may be the appropriate approach to pancreatic cancer cachexia management.

### Future directions

Current clinical management of pancreatic cancer cachexia is limited. None of the available therapies have shown lasting effects on weight stabilization and improvement in survival. Development of effective treatment for this disease remains a challenge.

Recent studies have focused on targeted therapies with anti-inflammatory properties (Table 4). IL-6 is a promising target, but many of the studies involving IL-6 antibodies have been in patients with advanced non-small cell lung cancer (NSCLC) and cachexia (Rigas et al., 2010; Schuster et al., 2010; Bayliss et al., 2011; Ando et al., 2013). Rigas and Schuster et al. reported a phase II randomized, double-blind, placebo-controlled trial with NSCLC patients evaluating the safety and efficacy of ALD518 (also known as BMS-945429), a humanized monoclonal IL-6 antibody, in treating cancer cachexia. ALD518 showed promising beneficial results. It increased hemoglobin levels and prevented loss of lean body mass (Rigas et al., 2010; Schuster et al., 2010). There was also a statistically significant improvement in fatigue score in the ALD518 group vs. placebo group that persisted over a 12 week period (Rigas et al., 2010). ALD518 was safe and well-tolerated (Rigas et al., 2010; Schuster et al., 2010).

**Table 4:** **Investigational drugs for the treatment of cancer cachexia**.

**ClinicalTrials. gov identifier**	**Title**	**Phase**	**Mechanism of action**	**Sponsor**	**References**
NCT01206335	Open label study with OHR/AVR118 in advanced cancer patients with anorexia-cachexia	II	Broad spectrum peptide-nucleic acid immunomodulator targeting cytokine production (including TNF-α and IL-6)	Ohr Pharmaceutical Inc.	ClinicalTrials.gov. Open label study with OHR/AVR118 in advanced cancer patients with anorexia-cachexia
NCT01433263	Clinical study BYM338 for the treatment of unintentional weight loss in patients with cancer of the lungs or the pancreas	II	Human monoclonal antibody against activin receptor type 2B (ACVR2B)	Novartis Pharmaceuticals	ClinicalTrials.gov. Clinical study of BYM338 for the treatment of unintentional weight loss in patients with cancer of the lung or the pancreas
NCT01505530	A phase 2 study of LY2495655 in participants with pancreatic cancer	II	Humanized monoclonal antibody against myostatin	Eli Lilly and Company	ClinicalTrials.gov. A Phase 2 study of LY2495655 in participants with pancreatic cancer

Another agent with anti-inflammatory activity is OHR/AVR118, a broad-spectrum peptide-nucleic acid immune modulator that targets both TNF-α and IL-6. A phase II study involving patients with advanced cancer and cachexia showed an improvement in anorexia, dyspepsia, strength, and depression (Chasen et al., 2011). A phase IIb study is currently ongoing to assess the efficacy of OHR/AVR118 in improving appetite and enhancing weight, lean body mass, strength, and quality of life (ClinicalTrials.gov. Open label study with OHR/AVR118 in advanced cancer patients with anorexia-cachexia). Further studies are needed to evaluate the safety and efficacy of these agents in patients with pancreatic cancer cachexia.

Myostatin and activin are involved in regulating skeletal muscle mass and function via the activin type IIB (ActRIIB) receptor. They inhibit myogenesis and the Akt/mTOR pathway involved in muscle protein synthesis and increase the expression of ubiquitin ligases to increase muscle degradation. Studies have investigated the therapeutic potential of inhibiting myostatin and ActRIIB in treating cancer cachexia. In pre-clinical studies, inhibition of ActRIIB prevented muscle wasting and prolonged survival in C-26 tumor-bearing mice (Zhou et al., 2010). BYM338 is a myostatin inhibitor developed by Novartis (Hanover, NJ, USA) to treat cancer cachexia. A multicenter, randomized, double-blind, placebo-controlled phase II trial is currently underway to investigate the efficacy of BYM338 in attenuating loss of body mass in cachectic patients with stage IV NSCLC or stage III/IV pancreatic cancer (ClinicalTrials.gov. Clinical study of BYM338 for the treatment of unintentional weight loss in patients with cancer of the lung or the pancreas). LY2495655 is another humanized antimyostatin antibody currently under investigation. A multicenter, randomized, double-blind, placebo-controlled phase II trial in patients with locally advanced or metastatic pancreatic cancer is ongoing to evaluate the efficacy of two different doses of LY2495655 in combination with gemcitabine in improving survival as well as lean body mass and physical performance (ClinicalTrials.gov. A Phase 2 study of LY2495655 in participants with pancreatic cancer).

## Conclusion

Approximately 80% of pancreatic cancer patients develop cachexia during the disease course and up to 30% die from cachexia-related complications (Fearon et al., 2006b; Bachmann et al., 2009). Pancreatic cancer cachexia is a multifactorial syndrome characterized by anorexia and hypercatabolism that are mediated by mechanical factors, pro-inflammatory cytokines, neuropeptides, hormones, and tumor-derived factors. In pancreatic cancer, energy homeostasis is compromised and oriented toward a continuous suppression of appetite and increased energy expenditure. This state leads to uncompensated loss of skeletal muscle and adipose tissue mass.

Further research is needed to elucidate the intricate mechanisms involved in the induction and maintenance of pancreatic cancer cachexia to aid in the development of future therapeutic targets. The management of cachexia remains limited but is currently an active area of research. The use of targeted immunotherapies have shown promising preliminary results. The future management of pancreatic cancer cachexia will likely involve a multimodal approach with nutritional support, combination agents and possible targeted therapies to improve quality of life, lean body mass, and even survival of pancreatic cancer patients.

### Conflict of interest statement

The authors declare that the research was conducted in the absence of any commercial or financial relationships that could be construed as a potential conflict of interest.
